# CRISPR and KRAS: a match yet to be made

**DOI:** 10.1186/s12929-021-00772-0

**Published:** 2021-11-15

**Authors:** Guzide Bender, Rezan Fahrioglu Yamaci, Bahar Taneri

**Affiliations:** 1 Institute for Molecular Cardiovascular Research, RWTH Aachen University Hospital, Aachen, Germany; 2 Faculty of Applied Natural Sciences and Cultural Studies, Ostbayerische Technische Hochschule, Regensburg, Germany; 3 Department of Biological Sciences, Faculty of Arts and Sciences, Eastern Mediterranean University, via Mersin-10, Famagusta, 99628 North Cyprus Turkey; 4 Department of Genetics and Cell Biology, Faculty of Health, Medicine and Life Sciences, Institute for Public Health Genomics, Maastricht University, Maastricht, The Netherlands

**Keywords:** KRAS, CRISPR, CRISPR-Cas, Genome editing, Cancer, Mutation, Colon cancer, Lung cancer, NSCLC, Pancreatic cancer

## Abstract

CRISPR (clustered regularly interspaced short palindromic repeats) systems are one of the most fascinating tools of the current era in molecular biotechnology. With the ease that they provide in genome editing, CRISPR systems generate broad opportunities for targeting mutations. Specifically in recent years, disease-causing mutations targeted by the CRISPR systems have been of main research interest; particularly for those diseases where there is no current cure, including cancer. KRAS mutations remain untargetable in cancer. Mutations in this oncogene are main drivers in common cancers, including lung, colorectal and pancreatic cancers, which are severe causes of public health burden and mortality worldwide, with no cure at hand. CRISPR systems provide an opportunity for targeting cancer causing mutations. In this review, we highlight the work published on CRISPR applications targeting KRAS mutations directly, as well as CRISPR applications targeting mutations in KRAS-related molecules. In specific, we focus on lung, colorectal and pancreatic cancers. To date, the limited literature on CRISPR applications targeting KRAS, reflect promising results. Namely, direct targeting of mutant KRAS variants using various CRISPR systems resulted in significant decrease in cell viability and proliferation in vitro, as well as tumor growth inhibition in vivo. In addition, the effect of mutant KRAS knockdown, via CRISPR, has been observed to exert regulatory effects on the downstream molecules including PI3K, ERK, Akt, Stat3, and c-myc. Molecules in the KRAS pathway have been subjected to CRISPR applications more often than KRAS itself. The aim of using CRISPR systems in these studies was mainly to analyze the therapeutic potential of possible downstream and upstream effectors of KRAS, as well as to discover further potential molecules. Although there have been molecules identified to have such potential in treatment of KRAS-driven cancers, a substantial amount of effort is still needed to establish treatment strategies based on these discoveries. We conclude that, at this point in time, despite being such a powerful directed genome editing tool, CRISPR remains to be underutilized for targeting KRAS mutations in cancer. Efforts channelled in this direction, might pave the way in solving the long-standing challenge of targeting the KRAS mutations in cancers.

## Background

In recent years, clustered regularly interspaced short palindromic repeats (CRISPR) based platforms have made a tremendous impact in biomedical research. These technologies have become essential genome editing tools, used for studying gene functions, for modeling diseases, and for developing novel therapeutic approaches [1–4]. Simply put, for disease purposes CRISPR-based technologies could be used to remove errors from the genomes.

Prior to the CRISPR applications, various genome editing tools such as zinc fingers nucleases (ZFNs) and transcription activator-like effector nucleases (TALENs), were introduced in cancer therapeutics [3]. Both of these methods work via their ability to customize their DNA-binding domains for recognition of the desired sequence. Their efficiency is profoundly dependent on the DNA-binding specificity of the major components, being zinc fingers and TALE proteins, respectively [5].

Despite the great change ZFNs and TALENs have brought to genome editing, they have had their disadvantages, which have led to the search for better alternatives. For instance, fabrication of engineered arrays of zinc fingers have been challenging for many laboratories. Due to the fact that zinc finger nucleases can only function in dimers, it is necessary to pair them up for a specific nucleotide sequence recognition. The challenge however presents itself in engineering of the nucleases, which is only done by a number of laboratories. TALENs, as the follower of ZFNs, have a simpler design, where the designed sequences can recognize individual sequences by discrete nucleotide matching. This gives an advantage to TALENs over ZFNs, in terms of design simplicity. However, the DNA consists of well conserved TALE repeats, and overcoming this to achieve better specificity might require off-base cloning methods. Even when this is possible, vastly repetitive TALEN sequences might cause another difficulty in the process of delivery via viral vectors [6–8].

CRISPR systems are based on base-pair matching of edited RNA and target DNA [6]. These platforms have been shown to be more adaptable and functional and are suggested to provide longer lasting therapeutic effects [9–11]. Compared to the prior genome editing tools, CRISPR systems have more flexibility and efficiency in terms of their ability to target a specific nucleotide sequence.

CRISPR-Cas systems are of several types. They naturally exist in about 90% of archaea and in about 40% of bacteria. They are used by these organisms as an immunity mechanism [12]. Specifically, they are parts of the adaptive immune system, which is a memory-based subsystem of the immune system. Following an initial response to a pathogen, adaptive immunity generates pathogen-specific recognition of the previously introduced pathogens and protects the organism from a secondary infection [13].

CRISPR is a set of repeated nucleotide sequences, found in the DNA of prokaryotes. These repeated nucleotide sequences are within a region of the genome, titled the CRISPR locus. Within the CRISPR locus, there are trans-activating CRISPR RNA (tracrRNA) gene, Cas gene and CRISPR repeats, which are essential elements of the system. Cas proteins are a family of nuclease enzymes that are responsible for cleaving the target DNA strand [14]. CRISPR RNA (crRNA) and tracrRNA fuse together to form a complex called the guide RNA (gRNA), in which the tracrRNA functions as a handle for Cas proteins and crRNA as the guide to the target site.

When foreign DNA is introduced, the CRISPR system enables the fusion of short DNA sequences from the virus into the CRISPR locus of bacteria. These short viral DNA sequences are called spacers and are stored in the CRISPR clusters. CRISPR sequences are transcribed into small pieces of RNA, called crRNA, which work together with the Cas proteins to form interfering complexes. These complexes base pair with the foreign invading DNA and cleave these sequences. When the virus is introduced to the organism again, the spacers can match the identical short sequences on the foreign viral DNA for recognition. Each time a new sequence is integrated into the CRISPR loci, the previous one is separated by a palindromic repeat sequence. These repeated sequences are recognized by the CRISPR-RNAs (crRNA). Proteins synthesized by the Cas gene form complexes, to which the crRNAs are later combined. These proteins help detect the nucleic acids of the target sequence [4, 15–18].

There are various types of CRISPR-Cas systems, categorized based on the use of different RNP complexes [19]. Mainly, CRISPR-Cas systems are sub-categorized into 5 types being Cas1, Cas2, Cas3, Cas9 and Cas10 [20]. In addition to these, there are novel systems that are being introduced such as spCas9-NG, base editing, xCas9, Cpf1, Cas13, and Cas14n [21].

CRISPR/Cas9 (CRISPR associated protein 9) is the most commonly used CRISPR tool and it is composed of two components, which are; a guide RNA (single guide RNA-sgRNA or gRNA) and the Cas9 subtype of Cas proteins. CRISPR sequences work as a guide for the Cas9 enzyme by forming a complex to recognize and cut the complementary sequences. This way, the gene of interest can be inserted at the cleaved and removed site [4, 22–24]. Following the specific target site, there is a sequence called the protospacer adjacent motif (PAM) which is a component of the invader’s DNA and not of the host's. The PAM sequence therefore helps distinguish the foreign DNA from the self DNA, which is required for the successful binding and cleaving by Cas nucleases [25, 26].

Cas9 protein, which is the most commonly used Cas protein version, is originally derived from the bacterial species *Streptococcus*. Cas9 protein has the ability to cleave the double stranded DNA at the sequences matching the guide RNA sequence. The gRNA works together with tracrRNA. Together they form a complex that is responsible for recruiting the Cas9 protein. These 2 RNA molecules, crRNA and tracrRNA, alongside the Cas9 protein are required for recognition of the foreign DNA leading to its cleavage by  Cas9.

CRISPR-Cas9 has been applied to mammalian cells as a genome editing tool aimed for therapeutic purposes. In mammalian systems, it has been used to edit causal mutations in monogenic disorders. It has become a widely used gene therapy tool applied both in cell lines and in animal models [2]. Cas9 has specifically been the most widely used version of the Cas endonucleases in mammalian systems due to its potential to target about 40% of human exons [27]. This technology has broad applications in animal cell lines and animal models, in specific human cancers are being targeted with CRISPR systems, using animal models [28]. A 2021 review by Komor et al. provides details on work describing the use of CRISPR systems, in Rett Syndrome, liver diseases and lung cancer, illustrating the wide range of applications of this technology in mammalian models [29].

In order to improve the transcriptional repression activities of Cas9 a Krüppel-associated box (KRAB) has been added to dCas9 forming a fusion protein of dCas9-KRAB [30]. This modification has enabled significantly less signals compared to Cas9 alone. However, in some cases since the repression was not efficient enough further modifications using other transcriptional regulations were applied [31].

Cas13, another RNA-targeting CRISPR protein has emerged as an alternative to Cas9 recently. Unlike Cas9, Cas13 targets the RNA interference systems. It binds to and cleaves the RNA rather than DNA substrates. The main difference between Cas9 and Cas13 is that Cas13 lacks a DNase domain. CRISPR-Cas13 system has 4 subtypes: Cas13a, Cas13b, Cas13c and Cas13d. It is considered a safer alternative as it can change the phenotype through RNA intervention without altering the DNA [32, 33].

Furthermore, there is Cas12j, the most recently reported mini RNA-guided endonucleases isolated from phages [34]. Cas12j sequence has little similarity to other Cas systems. They are responsible for staggered DSBs and avoid unspecific ssDNA cleavage. The small size of the Cas12j could overcome the problems of packing of the CRISPR systems for viral delivery [35].

It should also be noted that very recently in 2021, miniature CRISPR-Cas systems have been engineered. These systems, referred to as CasMINI, are more compact in size compared to the generally used CRISPR systems. Their efficiency in editing has been reported to be comparable to the current systems [36].

### Challenges of CRISPR technologies

CRISPR technologies have some limitations that keep them from being translated into clinical use. One of the major challenges has been toxicity in the process of delivering the Cas9-nuclease producing genes and directing RNAs into specific cells. The toxicity can occur during the introduction of CRISPR induced double-stranded breaks (DSB) that often triggers apoptosis. CRISPR induced apoptosis remains a safety concern that needs to be addressed when gene editing via these systems are being considered [37].

There are different delivery systems that are being examined, most common ones being the viral systems [37]. In a viral delivery system, vector selection is highly critical for an efficient process. Although they are commonly used they have been highly associated with limitations like carcinogenesis risk, restricted size for insertion, immune responses, as well as challenges in production in large-scales. For example Adeno-Associated Virus (AAV) as a vector for delivery has been the most commonly used option. However, AAV may not be the most preferred way of delivery since large-sized Cas9 systems may not be efficiently packed in them, thus decreasing the efficiency of gene transduction. Apart from the viral delivery systems, non-viral systems are being investigated for better transduction such as lipid- or polymer-based nanocarriers [4, 38, 39].

Recently, another significant challenge of CRISPR/Cas9 systems has been reported. In 2020, Enache et al. have shown that Cas9 expression in human cell lines could lead to emergence or expansion of inactivating TP53 mutations. They have demonstrated that TP53 is one of the top 4% genes to be mutated upon Cas9 introduction. Further, they reported that Cas9 expression specifically selects for p53-inactivating mutations. Therefore, the authors recommend investigation of p53 status of cells, after the introduction of Cas9 into TP53-wild type cells [40]. Hence, when considering CRISPR-Cas based genome editing, it would be critical to confirm the presence of functional p53 before and after engineering.

Furthermore, off-target effects of the CRISPR/Cas9 technique on the genome have raised big concern. Any possible modifications on the parts of the genome, which are not aimed to be altered, can result in undesired effects or complications in the long run. Minimizing the risk of off-target editing can be controlled through optimization of the Cas9 enzyme concentration and the expression time. However, this does not completely eradicate the possibility of off-target editing, which are essentially unwanted and unintentional mutations introduced during the editing process. Many off-target effects in human cell studies have been reported as insertions, deletions and point mutations. Apart from these mutations, there is a plausibility of sgRNA mismatching, due to the target sequences being short. The larger the genome, the higher the risk for the sgRNA strands to complement on off-target sites [1, 4, 16]. Studies have shown the existence of two different types of off-target effects. (i) ones due to the sequence homology of the target and (ii) ones that occur  anywhere in the genome. Off-targets are responsible for large deletions and genomic rearrangements, but also lead to lethal mutations causing loss of gene function especially in cancer cells [41, 42]. Although the use of CRISPR applications in clinical trials are increasing, possible off-target effects should also be addressed.

These off target effects could result in phenotypic alterations that are a series of events caused by unintentional binding of Cas9/gRNA complex at unwanted sites leading to DNA cleavage promoting editing and thus further regulatory changes in the genome. The off-target events due to Cas specificity, and this depends on several factors such as the host cell type, dosage and timing of Cas administration and specific genomic loci [43].

CRISPR/Cas systems have recently become widely-used in research for generating potential therapeutic tools, for single gene disorders such as cystic fibrosis and muscular dystrophy; infectious diseases like HIV and malaria, as well as complex diseases such as cancers. Many studies on cancers, using CRISPR/Cas systems, aim to target the inactivated tumor suppressor genes or overactive oncogenes, which are the major tumor promoting events [9, 44, 45].

Existing therapies fail to be sufficiently effective for most cancer types. Cancer is a very complex disease and difficult to treat mainly due to acquired resistance to the given drugs, and the detrimental side effects of the therapy, which significantly reduce the quality of life for patients. Search for personalized and rather sustainable therapy approaches for cancers have been unceasingly ongoing for decades. Due to the lack of better alternatives, long existing treatment methods for cancer are still widely in use, despite their long-lasting detrimental and even lethal side effects. Conventional methods like chemotherapy and radiation therapy are the most commonly and easily utilized therapy options even today. Systemic chemotherapy and radiotherapy aim to treat the tumor; they often fail since they cause damage in other organs or cause impairments in the immune system, which often shortens the life expectancy [46, 47]. On the other hand, rather new options such as immunotherapy have also proved to be undependable in the clinic, as they are not sufficient alone at later stages of the cancer, where chemotherapy is often required to be combined with [48]. Another promising yet challenging approach is molecular targeted therapy. The biggest obstacle here remains the identification of targetable molecules. When considered as personalized therapy, this becomes significantly challenging, due to the variety of driver mutations in each patient, let alone in each cancer type. Tumor heterogeneity is another difficulty in targeted therapy. Cancer cells could evolve and give rise to different genomic aberrations within the same tumor tissue. Different genetic and epigenetic backgrounds of the cell populations co-existing in the tumor area generate a highly challenging obstacle in terms of utilizing effective molecular targets and prevention of drug resistance [49, 50].

CRISPR/Cas systems are being researched as potential cancer mutation editing options. To date, there are over 6000 publications covering CRISPR applications on cancer. However, this number drastically comes down to about a hundred when specifically CRISPR applications on KRAS mutations are searched for (PUBMED Search dated 29/10/21). The KRAS oncogene, which is mutated in 15–30% of lung cancers, in 90% of pancreatic cancers and in 30–40% of colorectal cancers is one of the main driver mutations in these severe cancer forms, which are causes of significant public health burden and mortality [51–54]. This makes KRAS an essential target for all these three cancer types. However, to date, regardless of the tremendous efforts, KRAS has been untargetable using molecular targeted therapy approaches. CRISPR provides a novel molecular opportunity for editing KRAS mutations in cancer cells. In this review, we address the current status of CRISPR applications for editing KRAS mutations and for editing mutations of KRAS-related molecules, in these three specific cancer types.

### CRISPR applications in clinical trials of lung, pancreas and colon cancers

Although CRISPR has been tested in the editing of genes responsible for various diseases, its application in cancer at clinical level is still new. Based on the information on USA and EU-based clinical trial databases only one Phase I clinical study using CRISPR on 12 lung cancer patients has been accomplished (NCT02793856). In this study, programmed cell death protein 1 (PDCD1) gene was knocked out in T-Lymphocytes using CRISPR/Cas9 and expanded ex vivo to be infused back to the patients. The study showed safety and feasibility of application as well as its efficacy. Clinical trials on pancreas and colon are still recruiting patients for Phase 1 studies (NCT04426669, NCT04842812). However, another clinical trial on pancreatic cancer has been terminated by the sponsor (NCT03681951). As these trials are at early phases and as they are still ongoing, data, if any, on the effects of off-targets are not yet available.

## KRAS challenge in cancer

### The KRAS oncogene

**Fig. 1:**
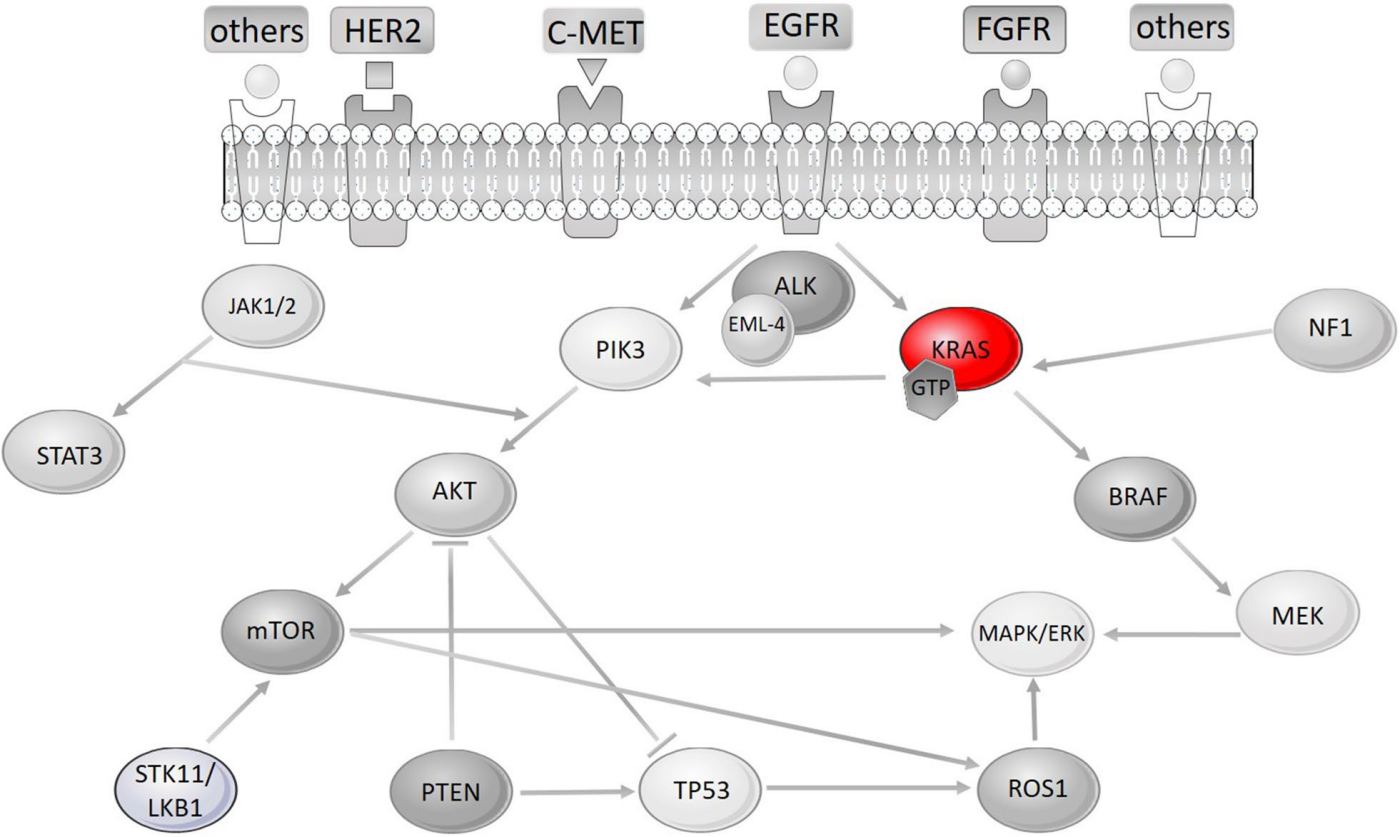
An overview of upstream and downstream effectors of KRAS. (Figure generated using SMART Servier Medical Art tools—https://smart.servier.com/.) Revised and uploaded with the manuscript

Kirsten rat sarcoma viral oncogene homolog (KRAS), is an oncogene, located on chromosome 12. It is a member of the Ras family, which consists of 2 other oncogenes, namely NRAS and HRAS. Proteins produced by Ras oncogenes are involved in cell differentiation, cell growth, cell proliferation, cell survival and apoptosis. Ras molecules are downstream of growth factors and upstream of pathways critical for cell proliferation and cell survival, such as MEK-ERK-RAF and PI3K-AKT-mTOR [55], as shown in Fig. 1.

KRAS codes for a protein involved in modifying transductive signals from the cytoplasm into the nucleus. Under normal physiological conditions KRAS is bound to guanosine diphosphate (GDP) and is in an inactive state. However, binding of KRAS to guanosine triphosphate (GTP) causes its activation. This process is involved in cell growth, proliferation and survival. In the event of a mutation, GTP is preserved, causing the signal to be constantly active, which results in higher amounts of cell growth and proliferation, as well as prolonged cell survival [56].

Although targeting of Ras oncogenes has been intensively studied, the market is not rich in effective inhibitors. Recently, a cautious optimistic outcome was presented regarding the testing of covalent mutant-specific inhibitors on human patients, generating a potential for direct targeting of KRAS in clinical trials [57–59]. However, problems still do exist, since only the KRAS G12C mutant can be targeted. In addition, the inhibitors are yet to pass through all of the clinical trial phases. Mutated KRAS can cause many changes in a significant molecular web covering several pathways, both as an inhibitor and as an activator of key molecules for tumor formation (Fig. 1). This crucial role of KRAS and the changes it causes on a bigger scale, makes it an attractive and necessary target for cancer management. However, the same reasons bring a lot of complexity and difficulties in targeting this molecule.

Mutations in the Ras family are most frequently observed in lung, colon and pancreatic cancers [60]. CRISPR applications in these three particular cancer types, specifically targeting mutations in KRAS and its related molecules are elaborated below.

### Lung cancer and KRAS

Lung cancer accounts for the highest cancer related deaths worldwide. Most commonly occurring form of lung cancer is the non-small cell lung cancer (NSCLC), which accounts for 85% of all cases, and is followed by small-cell lung cancer (SCLC) at 15% [61]. The distinction between these subtypes are mainly based on the microscopic appearance of the tumor cells [52]. NSCLC is subdivided histologically into lung adenocarcinoma, squamous cell carcinoma and large cell carcinoma [62]. Lung cancer symptoms are hard to detect at early stages. Therefore, it is commonly diagnosed at later stages, in which the treatment is harder to implement. Survival rates for lung cancer are reported to be much lower than other highly prevalent cancer types, such as colorectal cancer and prostate cancer. Five-year survival rates are reported to be about 56% for patients diagnosed at earlier stages, where the tumor has not spread outside of the lungs. However, only about 16–17% of lung cancer cases can be diagnosed at an early stage. The 5-year survival rates drop as low as 5–6% for cases diagnosed at later stages [63, 64].

NSCLCs mainly contain mutations in the KRAS, EGFR, BRAF, PI3K, MEK and HER2 oncogenes, as well as structural rearrangements in ALK, ROS1 and RET. Besides these mechanisms, amplification leads to activation of oncogenes, such as MET and FGFR1. KRAS and EGFR mutations, as well as ALK rearrangements are the main oncogenic drivers in adenocarcinomas. Therapeutic strategies aim to inhibit key signalling pathways that contain these oncogenes [65, 66]. KRAS is reported to be the most frequently altered oncogene in the NSCLC subtype. Mutations in this gene are found in 25% of NSCLC tumors mainly [67] in codons 12 and 13 of the KRAS gene. The most frequent mutations are G12C, G12V, and G12D followed by G13C [55, 68]. These mutations are reported to be located on exon 2, corresponding to a GTP binding domain [69]. In majority of the cases, these are missense mutations in which there is an amino acid substitution. Replacement of glycine (G) leads to resistance of the GTPase activating proteins (GAPs), which would normally hydrolyse the KRAS-bound GTP to GDP. The inability of GAPs on the GTP hydrolysis in mutant KRAS leads to a constitutively active protein [70]. Eventually signaling pathways downstream of KRAS such as BRAF/MEK/ERK and PI3K/AKT/mTOR pathways, are constitutively activated [71].

**Fig. 2:**
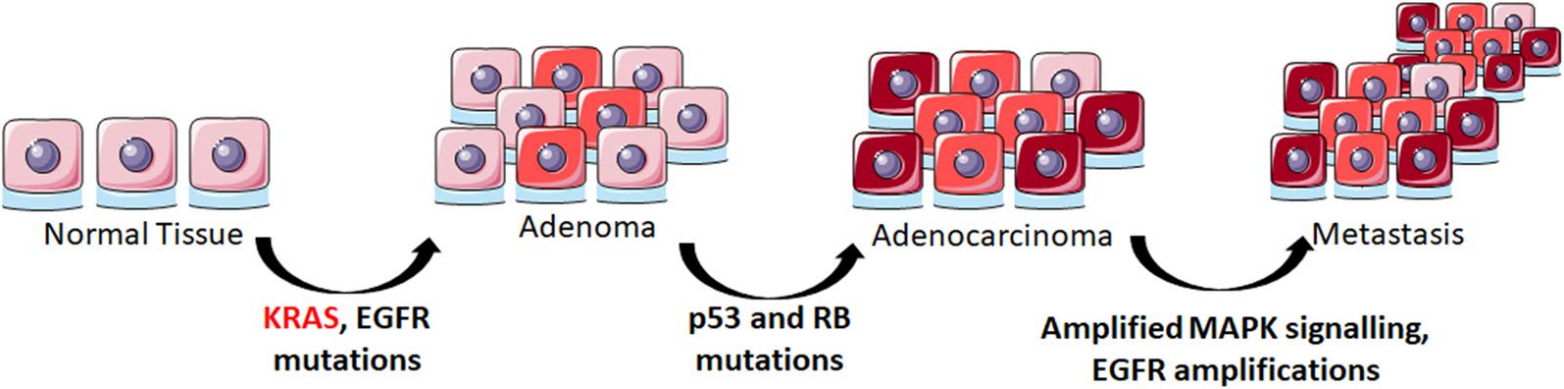
Involvement of driver mutations in the progression of lung cancer pathogenesis. An oncogenic Kras mutation is the initiator of tumorigenic progression in lung cancer, causing hyperplasia developing into adenoma. Mutations in p53 and RB contribute to carcinogenic switching of the tissue. Further amplification of MAPK signalling pathway elements, as well as amplifications in the EGFR gene result in metastasis to distant organs. (Darkening of the colours in the cells indicate progression of cancer stage.) (Adapted from Gazdar et al. 2008 and 2016 Feldser Laboratory [173, 174]) (Figure generated using SMART Servier Medical Art tools—https://smart.servier.com/.)

To date, effective direct targeting of KRAS in lung cancer has not been shown and remains a great challenge, as shown in Fig. 2 [55, 72]. The driver mutation of lung cancer pathogenesis is often determined to be in KRAS. One of the most common mutations (G12C, G12V, G12D and G13C) even on a single copy, is sufficient to initiate tumorigenesis. Very recently initial results of Sotorasib, a KRAS G12C inhibitor, in lung, colorectal and other types of cancers have been published [73]. A total of 129 cancer patients with KRAS G12C mutation who received heavy pretreatment joined the Phase I trial (NCT03600883) which showed the safety and efficiency of Sotorasib. Interestingly, 32% of lung cancer patients demonstrated a confirmed response, whereas only 7% of the colorectal cancer patients had a confirmed partial response. It has been hypothesized that this inconsistency in the tumor response could be due to KRAS G12C not being the dominant oncogenic driver in colorectal cancer [74].

EGFR mutations also occur at early stages of tumorigenesis, whereas the EGFR amplifications are observed at later stages contributing to aggressiveness of the disease, thus metastasis. Ras–Raf–Mek pathway is involved in signaling downstream from EGFR leading to the growth of cancer cells and tumor metastasis.

### Colorectal cancer and KRAS

Colorectal cancers (CRC) are the third most common cancers in the world and cause the fourth most common cancer-related deaths after lung, liver and stomach cancers [75]. Although it is mostly encountered in the Western countries, lately, the tendencies started to change in the fast developing countries as well [76]. A precursor lesion of CRC, i.e. adenoma, arises from glandular epithelium and is characterized by dysplastic morphology and altered differentiation of the epithelial cells. In the USA, the prevalence of adenomas is approximately 25% by the age of 50 and doubles by the age of 70 [77].

Mutations in oncogenes, tumor suppressor genes and genes involved in DNA repair are responsible for the development of CRC. Based on the mutation, CRC can be classified as sporadic or inherited. Point mutations are responsible for 70% of the sporadic cases. Mutations in the adenomatous polyposis coli (APC) gene result in familial and sporadic forms of CRCs. However, since APC is a tumor suppressor gene, either both alleles should be mutated or mutation in one allele should be followed by further mutations for CRC onset [78]. KRAS mutations usually rarely appear in the early lesions of adenomas, but reach up to 50% of the cases once the lesion grows more than 1 cm. Furthermore, loss of heterozygosity or mutations in the p53 gene are also closely associated with adenoma-carcinoma transition [79].

**Fig. 3:**
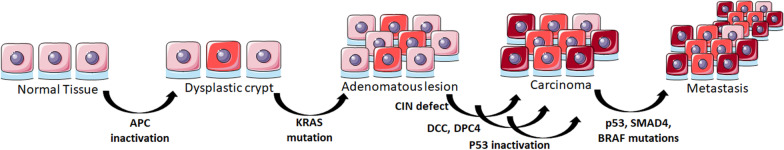
Schematic representation of CRC development upon occurrence of mutations. APC inactivation is responsible for the initial differentiation of the normal epithelial tissue into a dysplastic crypt. Further, mutations in KRAS lead to the formation of an adenomatous lesion, which could be followed by abnormalities in CIN, DCC, DPC4 and P53 that result in cancerous tissue in the colon. (Fig. 3 is adapted from Fearon et al., 2011 [79, 175]) (Figure generated using SMART Servier Medical Art tools—https://smart.servier.com/.)

Genomic instability is characteristic for CRC, which includes changes in chromosomal segregation, telomere dysfunction and DNA damage response, affecting critical genes such as APC and KRAS, which are involved in the maintenance of proper cell function [80]. Figure 3 shows a schematic representation for mutation accumulation during the evolution of normal epithelial tissue into CRC.

Mutations in the KRAS oncogene occur in approximately 40% of the CRC cases, majority being in exon 2 codon 12 [81]. These mutations are responsible for constitutive activation of MAPK, which promotes cell proliferation and contributes to the development of adenoma. However, these alone do not have a role in the initiation of adenoma, as shown in Fig. 3 [82].

KRAS is being used as a biomarker for CRC, mainly for treatment purposes. As in the other cancer types, presence of KRAS mutations cause resistance to tyrosine kinase inhibitor therapies leading to decreased overall survival and poor prognosis [83, 84]. Hence, development of KRAS targeting in colon cancer therapeutics is of essence.

### Pancreatic cancer and KRAS

**Fig. 4:**
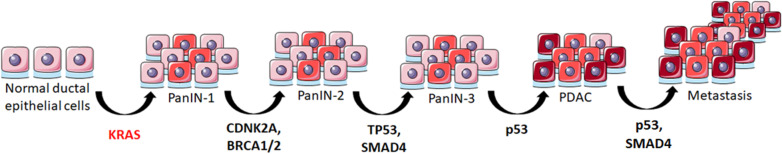
Schematic representation of evolution of ductal epithelial cells into Pancreatic Ductal Adenocarcinoma (PDAC). (Fig. 4 is adapted from Grant et al., 2016 [102, 103]. (Figure generated using SMART Servier Medical Art tools—https://smart.servier.com/.)

Pancreatic cancer is the third leading cause of cancer-related deaths and is associated with very poor prognosis [85]. The 5-year survival rate for pancreatic cancer is only 7–8% because of insufficiency of early detection methods and lack of symptoms [86]. Epithelial form of pancreatic cancer involves development of initially hyperplastic lesions, named as pancreatic intraepithelial neoplasia (PanIN). After acquiring a series of mutations, starting with KRAS, PanIN develops into Pancreatic Ductal Adenocarcinoma (PDAC) which makes up 90% of the pancreatic cancer cases [86], as shown in Fig. 4. PDAC is a very aggressive cancer form that is surrounded by a special fibro-inflammatory microenvironment, which contributes to cancer induction and growth [87]. Majority of the PDAC and early PanIN lesions involve mutations in the KRAS oncogene (92%) [88–91]. KRAS not only has an impact on the tumor, but also on its microenvironment. Its inactivation results in formation of chronic inflammation, by regulating an active stroma through the production of specific factors. These are namely Sonic Hedgehog (SHH), interleukin-6 (IL-6), and prostaglandin E that are expressed in a KRAS 7 dependent manner [87, 92, 93]. SHH is a member of the hedgehog signaling pathway, expressed by pancreatic tumor cells [94]. It functions in a paracrine manner [95] and activates hedgehog signaling in the stroma, regulating its maintenance [96]. IL-6 induces Stat3 activation in KRAS-mutant cells, which promotes PanIN progression and PDAC development in vivo [97]. It is important to note that IL-6 is secreted by myeloid cells that are recruited to the pancreas as a result of NF-κB induction, which is activated by KRAS G12D [98]. Chronic inflammation is associated with pancreatic cancer. Oncogenic KRAS mutations and the immune microenvironment may act synergistically to promote the development and progression of PDAC. KRAS activity is required at the early stages for the transformation to PDAC, since it is essential for the survival of precancerous cells [99].

Main function of pancreas is to maintain metabolic homeostasis by producing hormones that regulate blood glucose levels. However, this homeostasis is disrupted in tumor cells that possess an altered metabolism, named aerobic glycolysis, where glucose is extensively metabolized [100]. Interestingly, KRAS has also been shown to have a key role in metabolic reprogramming especially in glycolytic switch [101, 102]. Recently, mutant KRAS was shown to achieve this through increasing the expression of glycolytic enzymes. The increase in glucose uptake supports KRAS to promote synthesis of proteins, nucleic acids and fatty acids that are essential for pancreatic cancer cell proliferation [103]. Finally, mutant KRAS also regulates the transcription of key metabolic enzymes in the glutamine pathway, which is involved in the utilization of autophagy in PDAC. As evident, there is an extensive contribution of KRAS in the development and progress of pancreatic cancer, in addition to other genetic or epigenetic factors [99]. Hence, development of KRAS targeting in pancreatic cancer therapeutics remains of essence.

## CRISPR applications on KRAS

CRISPR systems, as a genome editing technology are currently being extensively tested for various biomedical purposes. One specific application of CRISPR is to turn off genes in cells or model organisms, relatively easily and quickly. The system has been tested in various forms to deplete the KRAS function in different cancer cells and animal models. As of May 2021, the most commonly used CRISPR technique, involving the CRISPR-Cas9 system, was used in 11 different studies to directly target KRAS mutations, all of which are shown in Table 1 and are detailed below.

**Table 1:** Direct targeting of KRAS in cancer via CRISPR systems (in chronological order)

CRISPR system	Cancer type	Targeted molecules	Cell type	Animal model	Publication year	Reference
CRISPR/Cas9	Lung	KRAS G12D G12C	A549, H23 (lung)	BalbC/nude A549 Xenografts	2018	[104]
CRISPR/SpCas9 and dCas9-KRAB	Lung	G12S	A549, H2228	NCG mice	2020	[105]
CRISPR/Cas9	CRC and Pancreas	G12V and G12D	SW480 (CRC) SW620 (CRC) SNU407 (CRC) AS-PC1 (PDAC)	n/a	2018	[106]
CRISPR/Cas9	CRC	G12V G12D G13D	HT29, SW403, SW480, SW620, LS513, and LoVo	BalbC nude	2018	[107]
CRISPR/Cas9	CRC	Whole gene knockout	SW480	BalbC nude	2020	[108]
CRISPR/dCas9/HDAC1	CRC and Lung	Whole gene knockout	HCT-116 (CRC), NCI-H358 (Lung)	n/a	2021	[109]
CRISPR/Cas9	Pancreas	Whole gene knockout	PDAC cells (PACO9 & PACO19)	Nude mice	2017	[110]
CRISPR-Cas13a	Pancreas CRC Lung	G12D	AsPC-1, PANC-1, HPAF-II, T3M4, LS174T, SK-LU-1	n/a	2017	[112]
CRISPR/Cas9	Pancreas	KRAS G12D	PDAC: Panc-1 and SUIT-2 (h) TB32047 (murine)	n/a	2019	[117]
CRISPR/CasRX	Pancreas	KRAS G12D G12C	n/a	Balb/C nude mice Orthotopic mice	2020	[120]
CRISPR/Cas9	Pancreas	KRAS G12D	FC1199, FC1242, FC1245 A9312 A9993	Orthotopic mice	2021	[122]

### CRISPR applications in direct targeting of KRAS mutations in lung cancer

To date, there have been limited reports on CRISPR applications in targeting KRAS in lung cancer. The first study was reported in 2018, where Kim et al*.* tested the system on lung cancer cell lines A549 and H23, both originating from NSCLC adenocarcinoma, in order to target the KRAS G12D mutation [104]. Genome editing was achieved with a chimeric single-guide RNA (sgRNA) that combined the crRNA and tracrRNA. Using purified Cas9 RNPs, authors showed that genome editing was more efficient with less off-target cleaves, than that would occur with plasmid- or viral-mediated delivery of Cas9 and sgRNAs. This was achieved by direct introduction of RNA or protein into the cells, without requiring additional steps, such as transcription and translation. Furthermore, authors showed that use of a low molecular weight protamine (LMWP), conjugated to Cas9, improved efficiency, precision and safety of the delivery system, since it caused self-assembly due to electrostatic configurations. Targeting of the mutant  KRAS using this system, resulted in inhibition of cell survival and tumorigenicity in vitro, as well as a decrease in tumor volume in xenografts. However, authors concluded that the evident technical complexity in the delivery of Cas9 RNPs could be challenging for future therapeutic use in human genetic diseases and that the system is needed to be tested in in vivo models prior to any clinical use.

In 2020, Gao et al*.,* for the first time, knocked out the KRAS G12S, using CRISPR/Cas9, as well as a transcription-regulating dCas9-KRAB system [105]. This novel system binds to the target sequence using dCas9 and downregulates mRNA transcription using the transcriptional repressor KRAB, which eventually inhibits the tumor growth in vivo. For this purpose, local injections of the AdV-Cas9-sgG12S system was performed on xenografts, where a 46% decrease in the tumor volume was recorded. When the mice were treated with the delivery system, a 30% decrease in tumor volume was observed. To test the specificity of the system mice were xenografted with A549 or H2228 cells, the latter with wild type KRAS. Authors showed that the effect was evident only in mice xenografted with A549, which contained the KRAS G12S mutant. Downstream molecules of KRAS were also affected by the CRISPR/Cas9 system G12S knockout. Phosphorylation rates of AKT and ERK were significantly decreased, which eventually led to inhibition of cell proliferation and growth arrest at S phase of mitosis. Furthermore, they screened  for 31,555 oncogenic mutations in the top 20 cancer driver genes selected from the Cosmic database, via high-throughput analysis, using different Cas9 variants [105]. They have shown that almost half of the investigated genes could be edited via one of the tested Cas9 variants. Authors concluded that efficiency of anti-tumor activity could be further improved by specific targeting of these mutations, and other oncogenic mutations.

### CRISPR applications in direct targeting of KRAS mutations in colorectal cancer

Various CRISPR systems have been used to target KRAS in colorectal cancer (CRC). One of these studies was conducted by Lee et al*.* in 2018, where the researchers introduced indels using the CRISPR/Cas9 system, causing frameshift mutations at the KRAS codon-12 sites using colorectal cancer cells (SW480, SW620, SNU407, AS-PC1 cell lines) [106]. For this purpose, they directly targeted KRAS c.35 G > T and c.35 G > A, resulting in G12V and G12D mutants. This disrupted the cell proliferation in cancer cells, but not in cells with wild-type KRAS. Since KRAS is essential for cell proliferation, indels were detrimental to the cancer cells that contained the specified KRAS mutation. In order to confirm this, the researchers transduced SW620, SW480, and HEK293T cell lines with Cas9-expressing lentiviral vectors with sgKRAS-G12V, or sgKRAS-WT. Viability of cells were then investigated using MTT proliferation assay, where a significant decrease in cell numbers was noted upon transduction with sgRNA targeting KRAS c.35 G > T. However, transduction of SNU407 and AS-PC1 with sgKRAS-G12D targeting c.35 G > A did not affect the cell viability negatively, since indel rates were very low, presumably due to cell line specific efficiency of transduction. The authors concluded that selectively reducing cancer cell proliferation using this new method could also be possible in vivo and could be a promising approach for cancer treatment [106].

In 2018, Kim et al*.* tested targeting the mutant KRAS, using CRISPR-Cas9 in various cancer cell lines (SW403, SW480, SW680, HT29, LS513, LoVo) and analyzed its effect on cell proliferation, survival and cancer growth in vitro and in vivo [107]. Initially, they managed to significantly downregulate mutant KRAS (c.35G > T (p.G12V), c.35G > A (p.G12D), and c.38G > A (p.G13D) in these cell lines, without affecting the wild-type KRAS. Indels causing in-frame frameshifts were found to be the cause of this downregulation in mutant KRAS cells. They further investigated whether this would have an effect on cancer cell survival, proliferation, and tumorigenicity in vitro. Survival and tumorigenicity was significantly inhibited in KRAS mutant cells (SW403, SW480, SW680, LS513), but not in HT29 cells with wild-type KRAS. In addition, authors tested the effect of mutant KRAS inhibition in vivo on immunodeficient mice, where animals were injected with KRAS mutant or KRAS WT cells subcutaneously. Upon disruption of mutant KRAS using Cas9 and sgRNA, a remarkable reduction in the tumor size (7, twofold) in KRAS mutant mice was determined. However, the authors mentioned that unwanted off-target oncogenic mutations can occur using the CRISPR system, despite at a very low rate, which could be below the detection limit of the system. In conclusion, Kim et al*.* suggested the use of CRISPR-Cas9 along with available cancer therapies, in order to improve their effects. Monotherapeutic use of CRISPR-Cas9 was not recommended, since the system does not function with 100% efficiency and could cause further mutations [107].

In 2020, Wan et al*.* used a new non-viral strategy using a supramolecular polymer (CP/Ad-SS-GD/RNP) to improve the delivery efficiency of the CRISPR/Cas9 system in CRC cells [108]. This supramolecule binds Cas9 and forms a stable nanoparticle achieving cell entry through endocytosis. In order to avoid the degradation of the nanoparticle in vivo, they covered its surface with an anionic polymer, hyaluronic acid. The intracellular degradation of CP/Ad-SS-GD was shown to release Cas9 RNP to execute its nuclease activity in vitro. Then, they used this system to knock out the mutant KRAS and analyzed the indels formed in the targeted sequence. Significant disruption of mutant KRAS was observed associated with decrease in proliferation and increase in Caspase-3 activity that is a marker for apoptosis. Therefore, the authors claimed that the system was a safe transduction method. Furthermore, the surface modification enabled selectivity of cancer cells, decreasing the proliferation and increasing the apoptosis, which eventually caused a significant decrease in the tumor size in vivo [108].

Very recently, in 2021, Liu et al*.* used a unique approach to silence KRAS via CRISPR technologies, which targeted the promoter region of KRAS [109]. In order to achieve this, researchers created a fusion protein by linking dCas9 and HDAC1 cDNAs in an expression vector that was transfected into the cells. Additionally, crRNAs were co-transfected that spanned the promoter region of KRAS. The aim was to deacetylate the promoter region of KRAS with the function of the HDAC1 and thus silence KRAS expression epigenetically. For this purpose HCT-116 (CRC), NCI-H358 cells (lung cancer) were used, where the KRAS expression was shown to be significantly decreased at RNA and at protein levels. The decrease was shown to be due to the binding of HDAC1 to the promoter. Furthermore, cell death was triggered in transfected cells, since colorectal and lung cancer cells are KRAS dependent. Authors then showed that the phosphorylation of downstream molecules, such as pERK and pAKT were also significantly decreased. Overall, they concluded that despite the high efficiency of epigenome editing, the delivery systems need to be further optimized [109].

### CRISPR applications in direct targeting of KRAS mutations in pancreatic cancer

CRISPR systems are being used to target KRAS in pancreatic cancer. In 2017, Muzumdar et al. became the first group to investigate the resistance mechanisms of KRAS inhibition and to uncover KRAS mediated pathways in pancreatic cancer via CRISPR [110]. Importantly, the sgRNAs of the CRISPR-Cas9 system used in this study could not distinguish mutant KRAS from the wild-type due to the absence of unique PAM sequences. In concordance with previous studies [111], only KRAS-dependent cells were vulnerable to KRAS knockdown, where cell viability was significantly decreased. The in-frame shifts caused by the indels, responsible for impaired proliferation of cancer cells both in vitro and in vivo, were confirmed using sequencing. These results once again showed the significance of KRAS in PDAC cell maintenance. However, it is well known that cancer cells develop alternative pathways to escape drug inhibition. In order to identify these bypass mechanisms, high-throughput drug screening was performed, where sensitivity to PI3K inhibitors was determined in KRAS deficient cells. Testing of several RTK inhibitors (against EGFR, FGFR etc.) showed that these could be sufficient, but not necessary for the PI3K activation in KRAS mutant cells, indicating the presence of compensatory mechanisms.

In addition, they investigated the resistance mechanisms upon KRAS inhibition. For this purpose, RNA sequencing was performed and gene signatures were analyzed. Results showed upregulation of genes associated with ribosomal biogenesis and protein translation, and downregulation of genes associated with interferon response and the metastatic cascade. A 16-gene signature with a prognostic significance in early stage patients with PDAC was developed. They showed that these KRAS knockout signatures were enriched in circulating tumor cells (CTCs) of PDAC relative to primary tumors. It is well known that CTCs display expression of genes downregulated by oncogenic KRAS. Based on this fact, authors claimed that decreased KRAS activity may promote gene expression changes that drive metastasis. In conclusion, the authors proposed simultaneous inhibition of KRAS and PI3K, as a viable combinatorial therapeutic strategy.

In a 2018 study, Zhao et al. tested the use of a novel Cas enzyme, namely Cas13a in a CRISPR system to specifically knockdown the KRAS G12D in pancreatic cancer cells, for the first time [112]. Cas13a was previously described to be a crRNA-guided RNA-targeting CRISPR effector that can bind and cleave a target RNA carrying a complementary sequence [113–115]. In order to efficiently knockdown the KRAS G12D several orthologous Cas13a proteins have been tested, where one of them reached 70% specific depletion of KRAS mRNA. Then the effect of CRISPR/Cas13a was tested on proliferation of pancreatic cells, where growth was significantly suppressed as well as apoptosis was elevated in KRAS mutant cells and tumors in vitro and in vivo. However, the authors noted that intratumoral injections in xenografts were necessary to achieve a significant effect, which is not a practical strategy for all types of tumors. Although the CRISPR/Cas13a system has previously been shown not to produce off-targets [116] this has not been assessed in this particular study. Therefore, more studies are needed to confirm the efficient use of the CRISPR/Cas13a system in the treatment of cancer.

In a 2019 study, Lentsch et al*.* tested the CRISPR/Cas9 system on Panc-1 (human), SUIT-2 (human) and TB32047 (murine) cells aiming to knock out KRAS [117]. Successful knockout of mutant KRAS G12D was achieved, leaving the wild-type intact. Presence of indel mutations were shown using sequencing methodologies, which caused significant decrease in the proliferation of KRAS mutant cells. Authors then analyzed the downstream signaling after KRAS knockout and found out that each cell line responded differently to the absence of KRAS. In brief, pErk was stable but pAkt, pStat3, pAMPK and c-myc levels were reduced in Panc-1 cells. On the contrary, Akt phosphorylation was lost but Erk phosphorylation was retained in SUIT-2 cells. Expression of pStat3 and pAMPK were observed only in one clone and c-myc was reduced in some other clones. Interestingly, pErk levels and the phosphorylation of Akt and c-myc remained constant in the murine TB32047 cell line, whereas pStat3 protein was only produced by two clones. Authors concluded that the results could be due to the organismal origin of cells and due to the unique evolution of each clone. Furthermore, differentially expressed genes (n = 417) upon KRAS knockout were also investigated. Mostly the down-regulated genes were those that are known to promote tumor cell invasion [118] or those that affect the energy supply of the cells [119]. Authors concluded that further studies need to be performed in order to investigate the interactions within different pathways upon mutant KRAS targeting.

In late 2020, Jiang et al*.* developed a new CRISPR/Cas 9 delivery system [120] which uses CasRx, a recently discovered Cas enzyme with high efficiency and specificity in genome editing [121]. In order to test the system, authors used pancreatic cancer cell lines PANC-1 (KRAS G12D), MiaPaCa2 (KRAS G12C) and normal pancreatic ductal epithelial cells H6c7. KRAS protein was found to be significantly reduced only in PANC-1 cells. However, CasRX-gRNA did not efficiently block the KRAS G12C mRNA expression in MiaPaCa2 cells.

Furthermore, pERK and pAKT were also decreased showing that the CasRx system was efficiently inactivating KRAS as well as its downstream molecules. In order to test the off-target effects of the system, RNA sequencing was performed. Authors showed that there was a fourfold decrease in the expression of mutant KRAS and KRAS related molecules, whereas only a limited number of genes were affected in wild-type KRAS expressing cells (H6c7). Functionality of this effect was also shown as a decrease in cell proliferation only in PANC-1 cells and corresponding xenografts but not in the MiaPaCa2 cells and respective mouse model. Furthermore, sensitivity to chemotherapy was increased tenfold and the tumor growth was suppressed in PANC-1 xenografts. They concluded that the CRISPR/CasRx system was efficient in blocking mutant KRAS G12D up to 90%. However, due to the heterogeneity of PDAC cells, therapeutic benefits of mutant KRAS silencing was not clear.

Finally, in 2021 Ischenko et al*.* have investigated the antitumor immune response of KRAS in pancreatic cancer [122] using premalignant and PDAC cell lines, as well as orthotopic mice. In this study, they aimed to understand KRAS dependency of pancreatic tumor cells, in terms of tumor progression and maintenance based on the cells’ tumorigenic capacity and immune suppression abilities. For this purpose, KRAS was knocked out using the CRISPR/Cas9 system and the efficiency was shown with deletions in the gene causing truncated non-functional proteins. Initially, KRAS KO cells and KRAS intact cells were injected to nude mice. In both cases tumors were formed, notably KRAS KO tumors developed much slower. Furthermore, tumor initiating cells in KRAS KO cells were almost half of that in KRAS intact cells.

In order to determine the cause of KRAS independence, authors analyzed cancer-related and DNA damage pathways. A transition to increased mesenchymal gene expression (epithelial mesenchymal transition-EMT) was noted, where SMAD4 was shown to compensate, at least in part, for the tumorigenic defects of KRAS KO in nude mice.

Later on injection of intact KRAS in syngeneic mice caused tumor formation and survival for 2 weeks. In the case of KRAS KO injection growth of tumors were completely inhibited, where survival was upto 8 weeks. More than 80% of these mice did not develop tumors even after 3 months. However, half of the cases that developed tumors evolved to metastasis, showing cancer progression which was independent of KRAS expression. In order to connect this finding to immunity, T cells were knocked out. They observed tumor formation similar to nude mice, leading to the conclusion that the immune system suppressed tumor formation in KRAS KO mice.

Overall, authors concluded that KRAS initiates cancer development, but at advanced stages immune system suppression takes over the role for tumor progression, thereby leaving the tumor KRAS independent. Even though tumor cells could survive without KRAS and still sustain their tumorigenic capacity, KRAS and its downstream molecules could play an essential role in immunosuppression of tumor formation and progression. Therefore, it is significant to both target the KRAS pathway and to promote antitumor immunity for an efficient outcome in PDAC treatment.

## CRISPR applications in KRAS related molecules

KRAS is involved in various pathways both as a downstream and as an upstream effector (Fig. 1). Therefore, a single change in the gene could cause dramatic changes in the flow of the pathways by resulting in activations or de-activations of other molecules. Genes that belong to the RAS family encode enzymes involved in hydrolysis of guanosine triphosphate (GTPase). This molecule connects the upstream modulators that are mainly cell surface receptors (i.e. EGFR and FGFR) to the downstream modulators, which are involved in proliferation and survival. Specifically, these are the RAF-MEK-ERK, PI3K-AKT-mTOR, and RALGDS-RA pathways [55, 123]. Hence, KRAS has a critical role in communication of cell surface receptors and cell proliferation and survival agents [72]. In addition, KRAS mutations often coexist with mutations in other genes. Therefore, these genes have also been subjects of targeted therapy research [124–126].

**Fig. 5:**
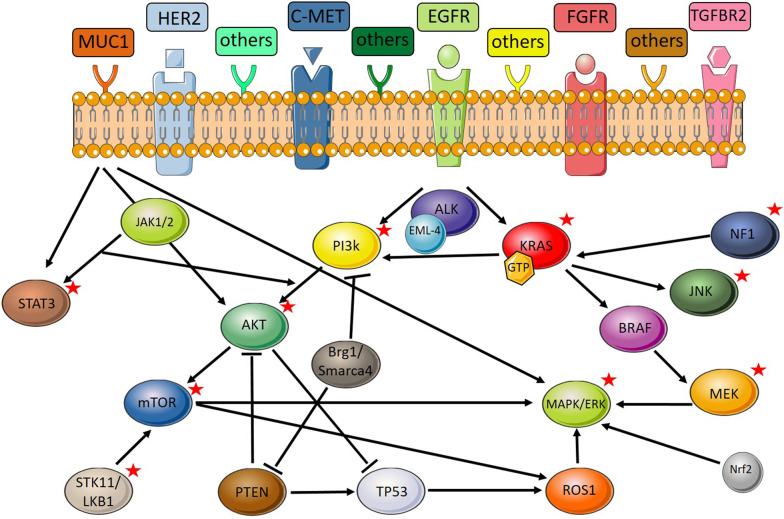
KRAS related pathways. Stars indicate molecules that have been targeted with the CRISPR systems. (Figure generated using SMART Servier Medical Art tools—https://smart.servier.com/.)

Figure 5 illustrates the KRAS related pathways, which details the molecules that have been targeted with the CRISPR systems. These molecules and the CRISPR research revolving around them are further detailed in this section.

In the recent years, CRISPR applications have been widely used to generate mutation-based in vivo and in vitro models, in order to study therapeutic approaches in KRAS-driven cancers, specifically in computational settings. Unfortunately, this technique like many others was found to be difficult to be directly applied on KRAS itself, as discussed above. An alternative approach is to target the molecules in KRAS related pathways. Several recent studies targeting the elements of KRAS molecular pathways using CRISPR are shown in Table 2 and are discussed below.

**Table 2:** Targeting of KRAS related molecules in cancer via CRISPR systems (information is provided first based on cancer type, then in chronological order.)

CRISPR system	Cancer type	Targeted molecules	Cell type	Animal model	Publication year	Reference
CRISPR/Cas9	Lung	MUC1-C	A549/KRAS(G12S), H460/KRAS(Q61H), H358/KRAS(G12C), H441/KRAS(G12V), H2009/KRAS(G12A), H1975/EGFR(L858R/T790M), PC9GR/EGFR(delE746_A750), H838s(mutant p53) and HEK293T	NCR nu/nu	2016	[127]
CRISPR/Cas9	Lung	KEAP-1	A549, H2030, H2009, KP1, KP2 and KPK	*Kras*^*LSL−G12D/*+^*; p53*^*flox/flox*^ (KP)	2017	[128]
CRISPR/Cas9	Lung	KEAP-1	NCI-H1299, HCC364, NCI-H1975, and HCC827	Nude mice HCC827 KEAP1^KO^	2017	[131]
CRISPR/Cas9	Lung	Smarca4 (Brg1), Arid1a and Setd2	HEK293 FT	C567B6/129Sv4: *Kras*^*LSL−G12D*^ and *Kras*^*LSL−G12D*^*;p53*^*flox/flox*^	2017	[132]
CRISPR/Cas9	Lung	MEK-ERK-PI3K pathways	HCT116, SW620, CRC119 and CRC240	NOD/SCID gamma mice or nude mice	2017	[133]
CRISPR/Cas9	Lung	PI3K pathway	HCT116, DLD1, CT26 and AALE	BALB/c mice	2017	[134]
CRISPR/Cas9	Lung	Utx	*Kras*^*G12D/*+^ MEFs, KP cells, KPU cells, and HEK-293 T	*Kras*^*G12D/*+^, *Trp53*^*L/L*^, *Lkb1*^*L/L*^, and *Utx*^*L/L*^ mice	2018	[135]
CRISPR/Cas9	Lung	MAPK7	MOR (ECACC), NCI-H2122 (ATCC), A549 (ATCC), NCI-H441 (ATCC)	*NCr nu/nu* mice	2018	[143]
CRISPR/Cas9	Lung	NF1	KP.1, KP.2, LKR10 and LKR13 mouse *KRAS*‐mutant/*TP53*‐WT/*NF1*‐WT (PDK), *KRAS*‐mutant/*TP53*‐WT/*NF1*‐mutant (PDKN1 and PDKN2) human	LSL‐*Kras *^*G12D*^; *Trp53* ^flox^ (KP) mice C57BL/6–129/Sv	2019	[138]
CRISPR/Cas9	Lung	STK11/LKB1 and SIK1, SIK3	A549, 634 T KP, VB-mouse lung tumor-derived KP and KPSik1 cells	Kras (KrasLSLG12D/ + ; R26LSL;luc/luc); KL (KrasLSLG12D/ + ; Lkb1fl/fl;R26LSL;luc/luc) KP (KrasLSLG12D/ + ; p53fl/fl;R26LSL;luc/luc) KPL (KrasLSLG12D/ + ; Lkb1fl/fl;p53fl/fl;R26LSL;luc/luc); *Sik1*^*fl/fl*^ and *Sik2*^*fl/fl*^ mice	2019	[139]
CRISPR/Cas9	Lung	STK11/LKB1 and SIK family	A549, H2122, H2126, H1650	*Kras*^*LSL*−*G12D*^, *p53*^*flox*^, *Lkb1*^*flox*^, *H11*^*LSL*−*Cas9*^ and *Rosa26*^*LSL*−*tdTomato*^ mice	2019	[172]
CRISPR/Cas9	Lung	PCDH7	HBECs, H1944	PCDH7 transgenic mice C57BL/6 J strain *Kras*^*LSL−G12D*^*; PCDH7*^*LSL/LSL*^; *Kras*^*LSL−G12D*^*; Tp53*^*fl/fl*^	2019	[141]
CRISPR/Cas9	Lung Pancreas	SHOC2	CFPAC-1, A549, NCI-H23, KP4, MIA PaCa-2, NCI-H2030, Panc 10.05, HCC364, NCI-H1299, HCT116, LOVO, PA-TU-8902, NCI-H1975, NCI-H2009, SU.86.86, MDA-MB-157, and MDA-MB-436	SCID Hairless Outbred (SHO) mice	2019	[142]
CRISPR/Cas9	Lung	EGFR	A549	n/a	2020	[144]
CRISPR/Cas9	Lung	STK11 and PTEN	n/a	n/a	2021	[151]
CRISPR/Cas9	CRC	Apc and Trp53	Lgr5 + stem cells murine AKP and human LS174	*Apc*^*fl/fl*^, *Kras*^*LSL−G12D/*+^, *Villin*^*CreER*^,*, Lgr5*^*eGFP−CreE*^, *Lgr5*^*CreER*^, *Rosa26*^*LSL−Cas9−eGFP*^ and *Rosa26*^*LSL−tdTomato*^	2017	[145]
CRISPR/Cas9	CRC	ERN1-JNK-JUN	HEK293	n/a	2018	[146]
CRISPR/Cas9	CRC	TGFBR2	HepG2 and HEK293T	NOD scid gamma (NSG) mice	2020	[147]
CRISPR/Cas9	Pancreas	PI3K	PACO9 and PACO19	*LSL-Kras *^*G12D*^*; p53 *^*flox/flox*^*; Pdx1-CreER, LSL-Kras *^*G12D*^*; p53 *^*R172H/WT*^*; Pdx1-Cre (KPC) and A13 clones*	2017	[110]
CRISPR/Cas9	Pancreas	GALNT3 and B3GNT3	SW1990, Capan1, NSP and SP	*Kras*^*G12D*^*; Pdx-1-Cre* (KC) and *Kras*^*G12D*^*; p53*^*R172H*^*; Pdx-1-Cre* (KPC)	2018	[148]
CRISPR/Cas9	Pancreas	ISG15	Panc02	C57BL/6	2019	[149]
CRISPR/Cas9	Pancreas	PRKD1	Panc-1, WI-38	PRKD1^KO^, KC, PRKD1^KO−^KC, p48Cre, LSL-KrasG12D and NSG	2020	[150]

### Targeting KRAS-related molecules in lung cancer via CRISPR systems

Lung cancer is a highly prevalent cancer type that very often harbours KRAS mutations. However, to date only a limited number of studies addressed KRAS-related molecules in KRAS-driven or KRAS harbouring lung cancer, specifically with the use of CRISPR/Cas systems. In this section, these limited numbers of studies are outlined.

In a 2016 study by Bouillez et al*.,* it was suggested that MUC1-C, a transmembrane protein induces the expression of MYC, which is known to be a hallmark of human cancers. CRISPR editing along with different techniques was used for targeting MUC1-C to suppress the MYC activity. Two types of KRAS mutant lung cancer cells, in specific A549 and H460, were used. MUC1-C was silenced in these cells using CRISPR genome editing, which suppressed MYC expression. Cells harboring EGFR mutations had little or no response in MYC regulation with CRISPR editing, suggesting the MYC and MUC1-C link can be specific to KRAS-mutant cells [127].

In a 2017 study, Romero et al*.* focused on indirect targeting of KRAS in a co-mutation setting in lung adenocarcinoma (LUAD). They focused on the KEAP1 gene, for which the loss of function mutations co-exist with KRAS mutations in 20% of LUAD tumors. CRISPR/cas9 was used to generate KRAS-driven LUAD mouse models to study the effect of the glutaminase inhibitors, which would target KEAP1 gene products. Based on their findings, they stated that combining genetic and metabolic approaches could be essential for identifying possible therapeutic targets. Loss of Keap1 was detected to contribute to further progression of LUAD through Nrf2 hyperactivation. Keap1/Nrf2 mutations have a dependency on glutaminolysis, hence making glutaminase an attractive therapeutic target. The glutaminase inhibition was shown to be suppressing tumor cell growth. A co-mutation seems to be necessary for an effective response to the given therapy. However, it is questionable how effective this kind of therapy can be on KRAS itself, considering the necessity of a co-mutation setting [128].

A similar approach to Romero et al. was conducted in a 2020 study published by Li et al*.* Epigenetic regulators of tumor immunity were screened with the use of CRISPR technology in in vivo models of KRAS-driven LUAD. They identified loss of the histone chaperone Asf1a to cause the tumors to be more susceptible to anti-PD1 treatment. Based on this, they suggested a new combination therapy targeting Asf1a, along with anti-PD-1 immunotherapy [129]. As a follow up, Li et al*.* published another study with focus on epigenetics. In a CRISPR based epigenetic screening, they have identified another histone chaperon, nucleophosmin 1 (NMP1). Genetic removal of NMP1 has been observed to result in decreased tumor progression, both in in vitro and in in vivo settings of NSCLC. In addition, tumor cells harboring KRAS mutations have shown NPM1 expression dependency. Therefore, loss of NPM1 changed the tumor cell metabolism and behavior, lessening the amount of tumor proliferating cells [130].

Many TKIs targeting the MAPK pathway have proved to be ineffective in the treatment of KRAS-driven lung cancers. In some patients, who have responded to the TKIs, there was development of resistance after a certain period of time. In order to investigate the acquired resistance of the MAPK signalling pathway, Wang et al*.* published a study in 2017 reporting CRISPR-Cas9 gene deletion screens. They identified that the loss of KEAP1 gene modulates the inhibition of BRAF, MEK, EGFR and ALK, which are important components of the MAPK signalling cascade. Through the loss of KEAP1, cell metabolism was changed to promote cell proliferation in the absence of MAPK signalling pathway. Authors emphasized the importance of KEAP1 gene and the possibility of alterations to this gene targeting a change in the TKI response in MAPK signalling [131].

In 2017 Walter et al*.* used CRISPR in an in vivo setting of KRAS G12D mutant mice and investigated the function of three main chromatin regulatory genes in lung cancer initiation and progression. They performed systematic inactivation of three genes namely, Smarca4 (Brg1), Arid1a and Setd2. They reported that the loss of Setd2 gene had initial tumor promoting effects. In the absence of Setd2, tumor progression has shown a rapid increase to almost 50%. On the other hand, loss of Smarca4 and Arid1a genes were shown to have stage-specific effects. Loss of both genes were effective at early stages of the cancer in terms of progression. Loss of Smarca4 in specific, was observed to effectively decrease the progression into later stages. However, this did not apply to Arid1a. Loss of Arid1a has, in contrast, contributed to the progression in the later stages. Based on these findings, they decided to run further analyses for Setd2 in human tumor samples and have come across a correlation between loss of Setd2 and poor survival. Authors concluded that Setd2 can potentially act as a tumor suppressor gene in lung adenocarcinoma, since its deficiency promoted shifting of the tumor grade [132].

In a 2017 study, Anderson et al*.* performed a CRISPR-Cas9 based systematic mapping of effectors in the KRAS pathway, in order to identify pathway associations for potential effective combination therapy. They addressed the MEK-ERK-PI3K pathways. A total of 70 screens were performed in models of KRAS driven cancers including lung, colorectal and pancreatic cancers via CRISPR/Cas9 based loss-of-function screening method. They identified tissue specific inhibitor combinations that target cell growth signalling, transcription, cell cycle and metabolism. They were able to successfully map tissue specific landscape of combination therapies for the pathways in which mutant KRAS is involved, which could help identify drugs that can work collaboratively. They recommended a combination of p38α/β inhibitor (LY2228820) and MEK/ERK inhibitors, which could be a potential sensitizing therapy for the lung and colon tissue [133].

Using CRISPR based screening in 2017, Martin et al*.* investigated the genetic dependencies of KRAS mutant cells needed for their growth and proliferation. Two isogenic cell lines were used for loss of function screening. They used a small hairpin RNA (shRNA) library in order to protect the essential genes, from off-target effects of CRISPR screening, loss of which could be toxic to the cells. They were able to identify multiple proteins of which the absence could reduce the growth of KRAS mutant cells selectively. Many of the genes were detected to be functioning in mitochondria. Additionally, in their in vivo studies, they have shown that mitochondrial inhibitors could decrease the KRAS-driven tumor growth [134].

In 2018, Wu et al. used CRISPR/cas9 technique to generate KRASG12D/ + mouse models, in order to screen a number of tumor suppressor genes. Through this screening, they identified 5 important tumor suppressor genes (Utx, Ptip, Acp5, Acacb, and Clu), which highly promoted lung tumorigenesis when knocked out and were associated with survival in lung cancer. Further in their investigations, they have confirmed the suggested role of Utx as an epigenetic regulator of tumor-suppression in a cross-model of Utx knockout and KRASG12D/ + model. They also identified that the tumor-promoting effects of Utx were mediated through upregulation of EZH2 levels. The EZH2 gene is a member of the Polycomb Group gene family and is involved in embryonic development. Knockout of Utx resulted in an increase in tumor progression. Following this information, EZH2 inhibitors were tested in vivo on KRAS-driven Utx knockout lung cancers and results showed Utx knockout models to be sensitive to EZH2 inhibitors [135].

In a 2019 study, Wang et al*.* investigated KRAS driven LUAD. Not far from NSCLC, 25% of LUAD cases are KRAS-driven and 3% of these have functional mutations in Neurofibromin-1 (Nf1). Nf1 is a regulator of GTPase RAS activity. Loss of Nf1 has been associated with the activation of RAS-MAPK pathway effectors, which are involved in oncogenesis. It is also noted that Nf1 protein is associated with the tyrosine kinase focal adhesion kinase-1 (FAK1) that is known to be serving in tumor progression [136]. Based on this, the research group suggested that FAK1 may have a significant role in KRAS-driven LUAD progression through loss of Nf1. Using the CRISPR/Cas9 platform Nf1 was silenced in the murine models. Effect of Nf1 loss and FAK1 hyperactivation on LUAD development was evaluated. Authors reported that Nf1 mutated cells are addicted to glutamine metabolism, due to the FAK1 hyperactivation, which consecutively activates the phosphoserine aminotransferase 1 (Psat1), an enzyme functioning as glutamine metabolizer. Glutamine metabolism is involved in many molecular pathways and acts as a redox balance in cancers. Glutamine metabolism adapts to changes in these pathways, oncogenes and tumor suppressors and therefore is often addressed in cancer therapies [137]. Loss of Nf1 has been associated with tumorigenesis acceleration in Kras-driven LUAD. Authors reported Nf1-mutated tumors to be addicted to glutamine. Based on these findings, they suggested that loss of Nf1 could generate susceptibility to Psat1 and glutaminase inhibitors. Hence, considering their effects upon the glutamine metabolism they have suggested Nf1 and Psat1 as possible therapeutic targets. Emphasizing on the potential role of Nf1 loss in resistance to BRAF and EGFR inhibitors, they also suggested this could be a potential strategy for other cancer types with related changes to the NF1-FAK1 pathway [138].

STK11 (LKB1) mutations are known to be very common in NSCLCs. STK11 is involved in mediating AMPK family kinases by producing a serine/threonine kinase that activates the kinases in the AMPK family. Salt-inducible kinases (the SIK family) belong to the AMPK-related kinases that are dependent on STK11 for their function. The SIK family is known for their activities in gene expression and transcriptional regulations. However, there is a lack of information on the function of many of these kinases’ in cancers. Hence, the function of STK11 through their activation is also not well understood. Based on this, Hollstein et al*.* studied the AMPK family kinases, using CRISPR in NSCLC cell lines and mouse models. Tumors that are Lkb1- (Stk11-) and Sik-deficient have shown similarities in terms of gene expression patterns and histology. This has suggested that they function along the same axis. Through their analyses, they identified an important role of the SIK subfamily, which functions in transcription. Loss of Sik1 and Sik3 in the SIK subfamily, resulted in intensified tumor growth in in vivo models of KRAS-driven NSCLC. Addition to this, they have shown loss of STK11 through loss Sik1/Sik3 can regulate the IL6/JAK/STAT pathway. Authors concluded that SIK1 and SIK3 were highly responsible for STK11 tumor suppressing functions and can therefore be important targets to mediate these pathways for therapeutic reasons [139].

Murray et al*.* published a similar study, in which they use CRISPR/Cas9 mediated genome editing to identify LKB1 (STK11) tumor suppressing properties in KRAS-driven lung adenocarcinoma mouse models. The kinase activity of the SIK family components are dependent on the LKB1. They have also identified the SIK family to contain potential therapeutic targets, as they are effectors of LKB1, as well as emphasized on the lack of mechanical knowledge on these components. Even though a few functional components of the SIK family are identified as possible targets with relatively less irrelevant functions, these components appear to be rarely mutated in human cancers, making them rather unappealing options. However, the main obstacle about the SIK family is their limited characterization, which prevents a clear understanding of what other components will be affected following a possible targeting in a bigger picture, considering their activities being influenced by various phenomena such as circadian rhythm, hormonal changes, and cellular depolarization [140].

The transmembrane receptor protocadherins (PCDHs) as a family are reported to have both oncogenic and tumor suppressing properties, whereas little is known about individual PCDH functions in cancers, as well as changes in their properties through gain or loss of function. Protocadherin 7 (PCDH7) is known to be often overexpressed in lung adenocarcinomas. Also, different levels of PCDH7 were associated with context-based functions in different cancer types.

Based on this, in their 2019 study Zhou et al*.* investigated the therapeutic potential of PCDH7 receptor through gain and loss of function in tumor cell surfaces. They identified that overactivation of PCDH7 has led to tumorigenesis in KRASG12D-driven lung cancer, and it had also triggered MAPK signalling. As opposed to that, inactivation of PCDH7 decreased tumorigenesis. Overall, they were able to determine oncogenic properties of PCDH7 in lung tumorigenesis by successfully applying CRISPR/Cas9 and using novel mouse models. Hence, inhibiting PCDH7 has been suggested as a critical strategy in lung cancer therapy [141].

MAPK signalling pathway is very critical in KRAS-mutated cancers and many inhibitors were developed to target the components of this pathway. Sulahian et al*.* have published a study in 2019, where they run a genome-wide loss-of-function CRISPR-Cas9 screening to identify the genes that can fit together with MEK inhibition. Screening was done using KRAS-driven pancreatic and lung cancer cell lines treated with MEK1/2 inhibitors (MEKi). They observed that some components of the RTK-RAS-MAPK pathway behaved as sensitizers to the given MEK inhibitors. Authors focused on a scaffold protein called SHOC2, which is a positive regulator of the MAPK. Degradation or suppression of SHOC2 were shown to be associated with disruption of survival pathways, recurrently signalling through the RTK pathway. Through their observations they concluded that SHOC2 could serve as a potential synthetic lethal target in combination with MEKi [142]. A similar strategy was followed in a 2018 publication by Dompe et al*.* They also focused on the MAPK pathway and potential therapeutic targets to be targeted along with MEK. Using CRISPR-Cas9 screening, they identified MAPK7 to be a strong candidate due to being involved in slowing down the MAPK re-activation like SHOC2. MAPK7 is also suggested as a part of a combination therapy with MEKi to eliminate possible re-activating agents’ activities for completely deactivating the MAPK pathway [143].

Tsukumo et al*.* have used CRISPR editing to examine the TKI effects on EGFR and KRAS co-mutated cells versus EGFR mutations and KRAS mutations alone in NSCLC. They suggested that EGFR-TKI can be an effective treatment option in cases harboring co-mutations. They used a KRAS mutated NSCLC cell line (A549) to generate co-mutation settings for testing this hypothesis. They generated and added different types of EGFR mutations with the CRISPR/Cas9 tool. They reported that cells containing EGFR mutations were more sensitive to the EGFR-TKIs than only KRAS-mutated cells. However, some EGFR mutations were reported to be more sensitive to TKIs than others, which can be a limitation based on their frequency. In addition to this, the same TKIs were not tested on EGFR mutated cells alone to compare the intensity of their sensitivity. Therefore the presence of KRAS mutation could be a negative factor in terms of EGFR sensitivity [144].

### Targeting KRAS-related molecules in colorectal cancer via CRISPR systems

Colorectal cancer is a highly prevalent cancer type that often harbours KRAS mutations. However, only a few studies addressed KRAS-related molecules in KRAS-driven or KRAS-harbouring CRCs, with the use of CRISPR/Cas systems. Here these studies in targeting KRAS-related pathways in CRC models are summarized.

Roper et al*.* have emphasized the lack of effective models to study CRC. In their 2017 study, they used CRISPR/Cas9 technology to generate in vivo CRC models by editing a selected group of commonly mutated genes including KRAS. They have edited the tumor suppressor genes Apc and Trp53 in epithelial cells of the colon via CRISPR/Cas9 system to promote tumor formation. They have also performed orthotopic transplantation of the Apc edited human and mouse organoids in the mouse models. With the orthotopic organoid transplantation they aimed to analyze activity of leucine-rich repeat-containing G-protein coupled receptor positive (Lgr5 +) intestinal stem cells in tumor progression and metastasis. They observed increased proliferation potential in the Lgr5 + stem cells in the colon following the transplantation indicating they have successfully generated sequential mutagenesis. Authors highlighted the convenience of the CRISPR/Cas systems in genome editing for generation of competent CRC models [145].

Sustic et al*.* conducted experiments aiming to unveil the genetic susceptibilities, which could be possible therapeutic targets for KRAS-driven colorectal cancer. Yeast were screened for genetic interactions between synthetic lethal and RAS mutations to detect these possible targets. Then, they used the CRISPR/Cas9 system to analyze the mechanistic intercommunications between these interactions, as well as downstream RAS signalling. For this purpose, they used KRAS mutated human colon cancer cells. They have identified Inositol-requiring enzyme 1 (IRE1), which is an endoplasmic reticulum (ER) stress sensor as synthetic lethal containing RAS mutations in yeast. ERN1, a human ortholog of the IRE1, was genetically removed from the KRAS-mutated colorectal cell lines. Removal of ERN1 did not appear to affect the tumor growth, but showed sensitivity against MEK inhibition in vitro. As opposed to that, a kinase inhibitor designed for ERN1 did not work together with MEK inhibition, which has led the group to investigate the ERN1 effect on MEK inhibitor responses. ERN1 knockout KRAS mutant colon cancer cells were screened for genes, of which the inactivation could contribute to a resistance to MEK inhibitors. They have identified various negative effectors of JNK/JUN signalling pathway that could be involved in this resistance. Based on that, the ERN1-JNK-JUN pathway holds significance in terms of sensitizing the KRAS-driven cancer cells to MEK inhibitors [146].

In a 2020 study, Michels et al*.* focused on the complexity of the tumor microenvironment. They have used the CRISPR/Cas9 system to generate organoids, with commonly occurring mutations and transplant these organoids into models to promote tumor growth. In addition, they coupled gRNA libraries with unique molecular identifiers (UMIs) that help amplify the reliability of the sequencing and can distinguish clone size. They screened for APC−/−; KRASG12D mutated human colon organoids for a library of tumor suppressor genes (TSG). Through this, transforming growth factor beta receptor 2 (TGFBR2) was detected to be the most frequently mutated TSG in APC−/–; KRASG12D models. Their findings point at the potential of TGFBR2 as a therapeutic target for the inactivation of the tumor growth promoting pathway [147].

### Targeting KRAS related molecules in pancreatic cancer via CRISPR systems

KRAS is very frequently commonly mutated in pancreatic cancers, the most common type being the PDAC. As discussed above, KRAS itself has been more of a subject to CRISPR/Cas mediated direct targeting studies in PDACs. Nevertheless, KRAS pathway related molecules are still of interest for indirect targeting.

In their 2017 study, Muzumdar et al*.* have focused on determining the effects of KRAS loss in PDAC, by looking into KRAS-regulated pathways. For this purpose, they have generated KRAS knock-down PDAC cells, using the CRISPR/Cas9 system. KRAS lacking cells and cells with KRAS have been screened with a group of compounds including kinase inhibitors, chemotherapeutic agents and epigenetic modifiers. None of the tested compounds had an effect on cells with KRAS. However, the KRAS deficient cells have shown enhanced sensitivity to pan-PI3K and mTOR inhibitors. Later, they looked into the interconnected pathways MAPK and AKT. PI3K inhibition caused an impediment in both of the given pathways concurrently, only in KRAS lacking cells. PI3K has long been treated as a RAS effector, but this study demonstrates that it could also act as an upstream regulator of RAS-MAPK pathways under certain conditions. Based on their findings, they suggested KRAS and PI3K targeting as a combinatorial approach for PDAC treatment [110].

There is an increasing amount of data suggesting the involvement of cancer stem cells (CSC) in tumorigenesis. A 2018 study by Barkeer et al*.* addressed the importance of the glycosylation process in pancreatic cancer stem cell (PCSC) mediation and evaluated the effects of its inhibitors on these cells. Glycosylation is the process by which the proteins and lipids are modified co- or post-translationally via glycans. As it is involved in many biological and cellular processes, including signalling, cell–cell interactions, protein folding, and immune response regulation, it is often associated with tumorigenesis and metastasis, when overactivated. Based on this Barkeer et al. have focused on GALNT3 and B3GNT3, which are enzyme-producing glycogenes, involved in the initiation of glycosylation. GALNT3 and B3GNT3 knockout SW1990 and Capan1 cell lines were generated using the CRISPR/Cas9 system to assess their involvement in PCSC regulation and further effects on metastasis. They observed a decrease in the stem cell transcription factors, as well as reduced colony formation and tumorsphere formation in the PCSCs, indicating a lower migration potential. Authors concluded that glycosylation plays an important role in mediating PSCSs and increased levels of GALNT3 and B3GNT3 help regulate this process [148].

In their 2019 study, Burks et al*.* have concentrated on the function of the interferon-stimulated gene 15 (ISG15), which is highly expressed in cancers, specifically PDACs. ISG15 belongs to the ubiquitin-like superfamily of proteins and is induced by interferons alpha and beta (IFN-α and IFN-β), which are known to be involved in inflammatory immune responses. Using the CRISPR/Cas9 tool, they generated ISG15 and UbcH8 (a conjugate enzyme for ISG15 protein) knockdown in Panc02 murine pancreatic cancer cells. ISG15 knockdown cells were subcutaneously injected to mice and the tumor size in wild type mice versus mice carrying ISG15 knockdown cells were compared. The same process was done using UbcH8 knockdown cells as a positive control. They have observed a significant decrease in the tumor volume in the ISG15 knockdown containing mice. Based on these results they concluded that ISG15 acts as a stimulator of tumor growth and therefore might have a therapeutic potential [149].

In a 2020 study, Armacki et al*.* investigated small extracellular vesicles (sEVs) and exosomes in a PDAC setting. sVECs and exosomes consist of lipids, proteins, RNA, DNA and are released by pancreatic tumors. Hence, they are thought to take part in tumor metastasis. Protein kinase D1 (PRKD1) is considered to be dysregulated in PDAC and is involved in inhibition of cell motility. The group has focused on PRKD1 activity in PDAC and effects of its dysregulation on sEVs production and release. They generated several genetic models via CRISPR/Cas9 system and small-hairpin RNAs (shRNAs). These models are, Prkd1 knockout mice (PRKD1KO mice) which is only specific for pancreas, mice expressing KRAS (KC mice), KC mice with Prkd1 knockout, as well as Panc1 cell lines (PRKD1KO-KC mice). The PRKD1 knockout cells were observed to produce sEVs with different contents compared to the sEVs generated by PRKD1 producing cells. The sEVs with altered content were subcutaneously injected into both mice models. They observed this application to result in increased metastasis to the lungs in PRKD1KO-KC mice. They suggested the lack of functional PRKD1 resulted in increased levels of sEVs with altered contents and that these sEVs could stimulate the tumor tissue to be more prone to lung metastasis in PDAC. Accordingly, they concluded that PRKD1 could serve as a potential therapeutic agent to prevent production of sEVs with altered contents and therefore promotion of metastasis [150].

In a 2021 study, Berthelsen et al*.* investigated the functions of main regulators of the mTor pathway, STK11 and PTEN, in KRAS-mutated lung cancer formation and progression. They used a CRISPR/Cas9 generated lung cancer mouse model with KRAS (gain of function) and Trp53 (loss of function) mutations (Trp53 − / − and KrasG12D). Again, using the CRISPR/Cas9 they tested the effects of Stk11 and Pten loss in this model and compared the loss of Stk11 to the loss of Pten. In this setting, they observed that loss of Stk11 (combined with Trp53 loss and Kras mutation) resulted in higher progression rates compared to loss of Pten. Based on this, the authors concluded that in KRAS-driven lung cancer, Stk11 mutations were essential for tumor progression. This also indicated that the co-occurrence of these 3 genetic alterations make a stronger base for tumor progression. Therefore, addressing one or two of these components could slow down or stop the progression. In addition to this, other regulators of the mTor pathway, which can alter the activity of Stk11, could be targeted for the same purpose. However, the authors noted that in human tumor samples, loss of Trp53 and Kras mutations happen to be mostly mutually exclusive, unlike the animal model, but Kras and Stk11 mutations were often seen to co-exist [151].

## Discussion and future directions

Cancer evolution is a multi-step process, often with tumor heterogeneity and KRAS mutations are seen in the early stages of this evolution process. KRAS is being used as a biomarker for cancers such as pancreatic, lung and CRC mainly for treatment purposes. Presence of KRAS mutations cause resistance to tyrosine kinase inhibitor therapies leading to decreased overall survival and poor prognosis. Hence, development of KRAS targeting in cancer therapeutics is of essence [83, 84].

Targetability of KRAS has proved to be troublesome. Due to many complications KRAS exerts on its microenvironment, its mutations are not only responsible for the progression, but also for the maintenance of the tumor. Unceasing efforts for direct targeting of KRAS have so far failed. This is due to the unresponsiveness of KRAS mutated tumors to the clinically available therapies. Since, KRAS is involved in a complex biochemical network communicating with many up- and downstream effectors, many attempts focused on unveiling the activation process of KRAS within this network of complexity. Treatment approaches focusing on targeting the KRAS effectors so far have not resulted in success as they proved insufficient to stall the mutated KRAS for an efficient outcome. Therefore, studies leaned towards a combinatory approach which again requires targeting KRAS directly for a full therapeutic response [140].

Introducing CRISPR platforms in cancer therapeutics seems to be a promising novel approach. As reviewed in this article, only a limited amount of studies have focused on targeting KRAS using CRISPR systems, leaving further limitations to be resolved prior to clinical application. Main challenges include (i) delivery efficiency, (ii) toxicity and (iii) off-target mutations. Firstly, delivery of the CRISPR-Cas complex to the target cells should be achieved. To safely and efficiently deliver Cas9 nuclease encoding genes and guide RNAs in vivo, a suitable vector is needed. As a viral system, AAV has previously been favoured as an option for gene delivery [152]. However, this delivery system may be too small to allow efficient transduction of the Cas9 gene. A smaller Cas9 gene could be used, but this has additional implications on efficacy [4]. A number of non-viral delivery systems are under investigation, such as plasmid based strategies [153], mRNA based delivery [154, 155], proteins, lipid nanoparticles [3] and physical methods like electroporation [156] and microinjection [157]. However, the gene delivery process requires further optimisation.

Secondly, toxicity of the delivery systems,which can occur in various forms, must be addressed. These are (i) DNA toxicity that is caused by CRISPR induced double-stranded breaks resulting in apoptosis [158] and (ii) immunogenic toxicity that is an outcome of pre-existing anti-Cas9 antibodies against the most commonly used orthologs [159]. In order to overcome these problems modifications of Cas9 variants should be tested intensively [37].

Lastly, another significant concern is the possibility of off-target effects on the genome. Unintentional edits of the genome could have profound long-term complications for patients, including tumor formation and malignancy via activation of oncogenes. Concentration of the Cas9 nuclease enzyme and the length of time Cas9 is expressed are both important to limit the off-target activity. Although recent modifications in the nuclease have increased specificity, further work is required to minimise off-target effects in order to establish the long-term safety of any treatment. Specificity and efficiency of the gRNA affects the cleavage by Cas9. Several factors such as the GC content [160], length of the gRNA [161], and chemical modifications [162] were shown to improve the specificity of Cas9. Therefore intensive efforts are exerted to reduce the off-target effects of the CRISPR technology [163].

Overall, many studies have suggested that CRISPR technology could be helpful in identifying therapeutic targets. However, application of the CRISPR technology in targeting KRAS mutations is still developing and has not been truly powerful in targeting KRAS itself. This is a common problem faced in other knockdown methods, which remains to be resolved.

Attention and focus mainly being put into the KRAS pathway molecules instead of KRAS itself is also indicating the difficulty of targeting KRAS with CRISPR techniques. KRAS driven NSCLCs do not harbor the same co-mutations in each patient. Therefore, time and cost effectiveness to identify which other molecules can be effectively targeted in the KRAS pathway is highly questionable. Five years after publishing our first review on KRAS in NSCLC, progress in targeted molecular therapy remains slow and only very recently, in 2021, a drug (Sotorasib) has received accelerated approval by FDA. Phase III trials on this drug are still ongoing on patients with NSCLC, given that they have already received one year systematic therapy [164].

KRAS oncogene is mutated in 15–30% of lung cancer cases, in 90% of pancreatic cancers and in 30–40% of colon cancers [51–54]. This makes KRAS an essential target for all these three cancer types. However, to date regardless of tremendous efforts KRAS has been untargetable using molecular targeted therapy approaches. CRISPR provides a novel molecular opportunity for editing KRAS mutations in cancer cells. In this review, we addressed in detail the current status of CRISPR applications for editing.

### Targeting collateral dependencies of KRAS mutations in cancer

As discussed above in detail, developing patient or mutation specific ‘to-the -point’ treatment methods for KRAS and related mutations have been challenging [165–167]. It is well known that cancer cells seek a way to survive, especially when their growth-dependent pathway is disturbed via drugs [168]. Therefore, non-mutational bypass mechanisms that arise after drug resistance should be defined for efficient therapy and better prognosable disease management. In 2019, Lou et al*.* announced their results of targeting collateral dependencies of the KRAS G12C mutant in lung and pancreatic cancer cells [169]. As a new terminology, the authors defined collateral dependencies (CDs) for the very first time in this study, as the ‘driver-limited cancer cell state’ whose inhibition could improve the effect of KRAS inhibitors. For this purpose, the authors tested the presence of molecules upstream and downstream of the KRAS G12C that act collaterally upon specific inhibition of mutant KRAS. The upstream CDs confirm the dynamic regulation of mutant KRAS, which was previously shown [170, 171]. This regulation was suggested to be cell-type specific, where different RTKs (e.g. EGFR, FGFR) played specific roles in signaling and activation in lung and pancreatic cancer cells. In the case of downstream CDS, complete inhibition was not achieved after targeting mutant KRAS; on the contrary, PI3K activity was maintained. Thus the authors concluded that KRAS is not the only element for cell cycle progression in tumor cells and simultaneous targeting of CDs, together with mutant KRAS, is essential for an efficient combination therapy [169]. Here, we would like to emphasize that this point should be carefully considered when selecting CRISPR targets for KRAS-driven cancers.

## Conclusions

Intensive efforts in finding a way to irreversibly silence KRAS mutations are yet to result in a valid therapy in various cancer forms with high public health burdens, including lung, colorectal and pancreas as reviewed here. Discovery of CRISPR/Cas systems has paved the way for new research directions in cancer, in terms of editing mutations. Studies highlighted in this review indicate a relatively strong therapeutic potential for the CRISPR systems in KRAS-driven cancers. In vitro and in vivo testing of various delivery methods, CRISPR gene editing units and dosage analyses produced promising results in the direction for further analyses and potential clinical applications. Hence, it would be of importance to improve this technology within a KRAS specific perspective. It should also be noted that technical challenges remain to be resolved prior to the potential use of CRISPR/Cas9 in treatments at the bedside. These include maintaining efficient and safe delivery, and achieving high specificity.

Nonetheless, based on the data analyzed here, we conclude that KRAS should be considered as a potent target for the CRISPR systems, for cancer therapeutics. Furthermore, we conclude that due to the collaterally dependent genes and their implications in the evolution of cancer, KRAS mutations should not be targeted alone, but together with related molecules in relevant pathways.

## Data Availability

Not applicable.

## Abbreviations

AAV: Adeno-associated virus
AKT: Protein kinase B
ALK: Anaplastic lymphoma kinase
AMPK: AMP-activated protein kinase
APC: Antigen-presenting cell
Arid1a: AT-rich interactive domain-containing protein 1A/ the gene producing it
Asf1a: Anti-silencing function 1A histone chaperone, a protein coding gene
BALBC: Bagg and Albino
BRAF: B-Raf proto-oncogene
Brg1: A transcriptional regulator, also known as ATP-dependent chromatin remodeler (SMARCA4)
Cas: CRISPR associated proteins
CasRX: Cas ribonuclease
CD: Collateral dependencies
CF: Cystic fibrosis
c-myc: Cytoplasmic-Myelocytomatosis oncogene
CRC: Colorectal cancer
CRISPR: Clustered regularly interspaced short palindromic repeats
crRNA: CRISPR RNA, contains various targeting sequences
CTC: Circulating tumor cell
EGFP: Enhanced green fluorescent protein
EGFR: Epidermal growth factor receptor
EMT: Epithelial mesenchymal transition
ERK: Extracellular signal-regulated kinase
EZH2: Enhancer of zeste homolog 2
FAK1: Focal adhesion kinase 1
FGFR1: Fibroblast growth factor receptor 1
GAP: GTPase-accelerating proteins
GDP: Guanosine diphosphate
GTP: Guanosine triphosphate
GTPase: Guanosine triphosphatease
HDAC1: Histone deacetylase 1
HER2: Human epidermal growth factor receptor 2
HIV: Human immunodeficiency virus
HRAS: Harvey rat sarcoma viral oncogene homolog
JAK: Janus kinases
KEAP1: Kelch-like ECH-associated protein 1
KRAB: Krüppel associated box
KRAS: Kirsten rat sarcoma oncogene homolog
K-Ras: Kirstan rat sarcoma protein
Lamb1: Laminin subunit beta-1
LKB1: Liver kinase B1
LMWP: Low molecular weight protamine
LUAD: Lung Adenocarcinoma
LY2228820: Ralimetinib
MAPK: Mitogen-activated protein kinase
MEK: MAPK/ERK kinase
MEKi: MAPK/ERK kinase-inhibitor
MET: Methoprene tolerant oncogene
mTOR: Mammalian target of rapamycin/mechanistic target of rapamycin
mRNA: Messenger RNA
MTT: 3-(4,5-Dimethylthiazol-2-yl)-2,5-diphenyl tetrazolium bromide
MUC1-C: Mucin1-C terminal
MYC: Myelocytomatosis oncogene
NCG: NOD CRISPR Prkdc Il2r Gamma
NF1: Neurofibromin 1
NHEJ: Non-homologous end joining
NPM1: Nuclear matrix protein 1
NRAS: Neuroblastoma RAS viral oncogene homolog gene
N-Ras: Neuroblastoma RAS viral oncogene homolog protein
NSCLC: Non-small cell lung cancer
PAM: Protospacer adjacent motif
PanIN: Pancreatic intraepithelial neoplasia
PcR: Polycomb group
PDAC: Pancreatic ductal adenocarcinoma
PD1: Programmed cell death protein-1
PI3K: Phosphoinositide 3-kinase
PRKD1: Protein kinase D-1
RAF: Rapidly accelerated fibrosarcoma
RALGDS: Ral guanine nucleotide dissociation stimulator
RET: Rearranged during transfection gene
RNP: Ribonucleoprotein
RTK: Receptor tyrosine kinase
ROS1: Reactive oxygen species 1 gene
SCLC: Small cell lung cancer
sEV: Small extracellular vesicles
SHH: Sonic Hedgehog
SIK: Salt-inducible kinases
sgRNA: Single guide RNA
shRNA: Short hairpin RNA
SHOC2: Leucine-rich repeat protein
Setd2: SET domain containing 2, histone lysine methyltransferase protein
siRNA: Small interfering RNA
Smarca4: SWI/SNF related, matrix associated, actin dependent regulator of chromatin, subfamily A, member 4/BRG1
STAT: Signal transducers and activators of transcription gene
Stat3: Signal transducers and activators of transcription 3 protein
STK11: Serine/threonine kinase 11
TKI: Tyrosine kinase inhibitors
tracrRNA: Trans-activating crispr RNA
TSG: Tumor suppressor gene
Utx: Ubiquitously transcribed tetratricopeptide repeat, X chromosome/Lysine-specific demethylase 6A
